# Clinical Characteristics of Patients with Type 2 Diabetes Mellitus Receiving a Primary Total Knee or Hip Arthroplasty

**DOI:** 10.1155/2019/9459206

**Published:** 2019-11-19

**Authors:** Annalisa Na, Laurie Jansky, Zbigniew Gugala

**Affiliations:** ^1^Department of Orthopaedic Surgery and Rehabilitation, University of Texas Medical Branch, Galveston 77555, USA; ^2^Division of Rehabilitation Sciences, University of Texas Medical Branch, Galveston 77555, USA

## Abstract

**Aim:**

The purpose of this study is to explore clinical characteristics of patients with T2DM receiving a primary knee (TKA) or hip (THA) arthroplasty to patients without T2DM receiving a TKA or THA and patients with T2DM with no history of osteoarthritis (OA).

**Methods:**

The study included a retrospective database review of 500 consecutive primary TKA or THA identified with ICD-9 codes and 100 consecutive T2DM patients. Patients who received a TKA or THA were screened for inclusion and exclusion and divided into with or without T2DM groups. A comparison group of patients with T2DM only without arthroplasty was screened to exclude patients with a history of OA or arthroplasty. All groups were compared based on demographic and relevant comorbidity differences. OA characteristics, including OA and previous arthroplasty of the involved and contralateral joints, were compared between patients with and without T2DM receiving a TKA or THA. Finally, patients with T2DM with and without TKA or THA were compared for T2DM differences.

**Results:**

Study results found that among those receiving a primary arthroplasty, patients with T2DM were more likely to be obese and older and reported cardiovascular, urinary, dyslipidemia, and peripheral neuropathy than those with T2DM. Among the T2DM individuals, those receiving an arthroplasty surgery were older and obese and more likely to report peripheral neuropathy; however, those with T2DM with no OA were more likely to report atherosclerosis and cardiovascular disease. Within the arthroplasty subgroup of individuals with T2DM, those requiring antidiabetic medication were 4.5 times more likely to have contralateral OA or arthroplasty.

**Conclusions:**

The results of this study suggest that patients with T2DM requiring a primary arthroplasty are a unique subgroup that requires careful considerations as they are often older, have obesity, and specific comorbidities predisposing to worse postoperative outcomes than their non-T2DM arthroplasty counterparts. Therefore, clinical practice and future studies must consider strategies that would limit OA and arthroplasty management delays while accounting for comorbidities and patient characteristics.

## 1. Introduction

A primary total knee (TKA) or total hip (THA) arthroplasty is an effective surgical intervention for improving function among patients with terminal osteoarthritis (OA) [1]. Unfortunately, arthroplasty management for patients with type 2 diabetes mellitus (T2DM) is often subjected to treatment delays due to underlying comorbidities [2]. Anticipated risks of postoperative complications, readmission, and extended hospital stay associated with arthroplasty patients with T2DM further contribute to delaying surgery. Hence, the postoperative management of T2DM arthroplasty patients is demanding and results in limited participation in postoperative rehabilitation care, which, in turn, compromises function.

Delaying arthroplasty surgery for T2DM patients with terminal OA can have negative medical and functional consequences, thereby threatening outcomes. Typically, these patients receive joint arthroplasty at a higher age and with more OA and more advanced and with multiple joint involvements [3]. The presence of diabetic neuropathy can mask the OA symptoms [4] and contribute to arthroplasty delays. On the other hand, the pain management strategy through limiting or avoiding mobility and weight-bearing activities can increase the risk for the physiological and medical decline related to physical inactivity [5]. This is especially disconcerting for patients with T2DM because limited physical activity can threaten glycaemic control and produce new or worsen comorbidities [6, 7]. Poor glycaemic control can also cause metabolic dysregulation and additional diabetic complications, such as metabolic syndrome, neuropathy, retinopathy, and nephropathy [8]. Exacerbation of comorbidities can be potentially life-threatening and pose a high risk of cardiovascular events [9].

Hence, understanding the underlying comorbidities and their effects on the OA to arthroplasty course and management among patients with T2DM would provide insight into determining more effective care strategies. The overall objective of the study is to retrospectively review T2DM patients with terminal knee or hip OA requiring arthroplasty as compared to their non-T2DM counterparts. Specifically, the purpose of this study is to explore patient-specific characteristic differences for patients with T2DM receiving a primary knee or hip arthroplasty, so that future studies can establish OA and arthroplasty algorithms that optimize outcomes.

## 2. Materials and Methods

### 2.1. Ethical Statement

The study protocol was reviewed and approved by the University of Texas Medical Branch Institutional Review Board.

### 2.2. Data Collection

We retrospectively reviewed charts of a cohort of 500 consecutive patients who received a primary TKA or THA at the University of Texas Medical Branch between 2008 and 2013.

**Figure 1:**
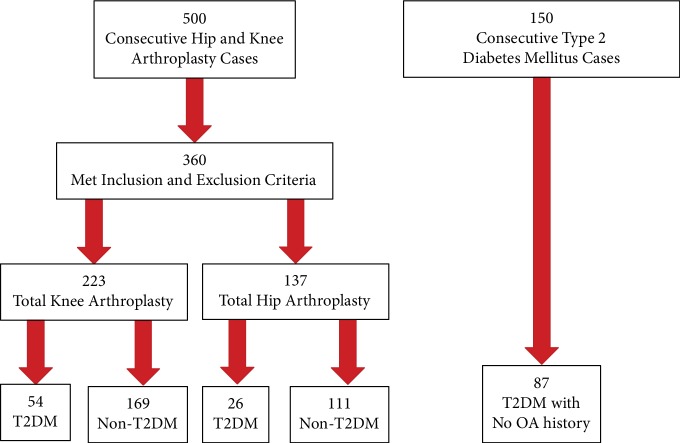
Chart review flow. Charts were screened for inclusion and exclusion criteria of patients receiving a total hip or knee arthroplasty withand without type 2 diabetes mellitus (T2DM) and patients with T2DM without a history of osteoarthritis (OA) or arthroplasty.

The International Classification of Diseases, Ninth Revision (ICD-9) codes were used to identify eligible patients. The 500 consecutive charts were then screened to meet inclusion/exclusion criteria. Patients with knee or hip arthroplasty due to fracture, posttraumatic arthritis, rheumatoid arthritis, developmental dysplasia of the hip, avascular necrosis, revision arthroplasty, hemiarthroplasty, or incomplete medical records were excluded. Patients who had bilateral or multiple arthroplasties during the designated timeframe were considered individual subjects for each arthroplasty. Patients, who met the *a priori* determined criteria were reviewed to extract patient characteristic, comorbidities, and, when appropriate, OA and arthroplasty and T2DM status. From the same University of Texas Medical Branch database, a separate chart review of 150 consecutive patients with the T2DM ICD-9 code, without OA or arthroplasty and admitted to the hospital for any reasons other than arthroplasty, were evaluated as a control T2DM group (Figure 1).

#### 2.2.1. Patient Characteristics

Patient characteristic data included age on the day of surgery, age on a specific date, sex, ethnicity, smoking status, and height and weight. Ethnicity was defined as Caucasian, Hispanic, Black, or Asian. Height and weight were used to calculate body mass index (BMI). Age on day of surgery allowed for comparisons of when arthroplasty occurred, while age on a specific date allowed for group comparisons with individuals in the T2DM only (i.e., no-OA) group.

#### 2.2.2. Comorbidities

ICD-9 codes were searched and identi- 2 fied for hypertension, dyslipidemia, nephropathy, urinary involvement, cardiovascular disease, coronary atherosclerosis, hypothyroid, and peripheral neuropathy. Type of peripheral neuropathy, date of when peripheral neuropathy was first noted, treatments of peripheral neuropathy, and time in years from the date of when peripheral neuropathy was first noted and date of arthroplasty surgery was calculated to determine peripheral neuropathy duration.

#### 2.2.3. Previous Osteoarthritis and Arthroplasty Status

ICD-9 and CPT codes for previous hip or knee OA and arthroplasty were recorded. Charts were further reviewed to determine if OA or arthroplasty history was of the involved or contralateral joints. Data and date of any previous hip or knee arthroplasty were also recorded.

#### 2.2.4. T2DM Status and Characteristics

T2DM was identified with ICD-9 codes for those who received a TKA or THA and for those with no history of OA. Additional T2DM data included the date of T2DM onset, hemoglobin A1c (HgbA1c), and anti-T2DM medication. For the T2DM and arthroplasty group, time of T2DM onset to surgery was calculated from the respective dates. HbA1c was extracted for the last measurement before surgery for those with T2DM with surgery and the most recent HbA1c at the time of clinical data retrieval. Any T2DM management not requiring medication management was documented (e.g., diet and exercise), and anti-diabetic medications were categorized by oral, insulin, or both.

### 2.3. Statistical Analysis

We used chi-square for nominal variables and one-way ANOVA with correction as needed for continuous variables to identify group differences. With mixed-models regression, we controlled for patient characteristics significantly different between groups and explored the relationships between comorbidities and T2DM or arthroplasty status. Variables that demonstrated significant group differences were included in radar plot(s). Vertices of radar plot represented group proportions of each variable, including patient characteristics, comorbidities, and OA and arthroplasty status for arthroplasty groups and patient characteristics, comorbidities, and T2DM status for T2DM groups. Continuous variables were presented on the radar plots using cut-offs reported in the literature. Specifically, older adults were defined as ≥68 years for TKA and ≥63 years for THA, as these age cut-offs were previously reported as predictors of inferior functional outcomes [10, 11]. For comparisons between T2DM groups with and without arthroplasty, older adult was defined as ≥60 years. Previous studies report that ages ≥ 60 years were an independent risk factor in T2DM-related complications [12]. Obesity was defined with a BMI cut-off of ≥30 kg/m^2^, which is a predictor of arthroplasty complications [13]. Inadequate glycaemic control was defined as HbA1c ≥ 7.5%. Goldstein et al. [14] reported strong relationships to peri and postoperative complications when prearthroplasty HbA1c ≥ 7.5%. Finally, we calculated the odds ratio to determine the relationship between contralateral OA or arthroplasty involvement and T2DM management.

## 3. Results

Of the 500 TKA or THA charts reviewed, 223 TKA patients and 137 THA patients met the inclusion and exclusion criteria (Figure 1). T2DM prevalence for the TKA group was 24.2% (*n* = 54) and 19% (*n* = 26) for the THA group (Table 1). Of the 150 T2DM charts reviewed, 87 charts included no history of OA or arthroplasty (Table 1).

### 3.1. Patient Characteristics

Table 1 summarizes group characteristics of individuals with and without T2DM respective to TKA or THA and those with T2DM and no-OA.

**Figure 2:**
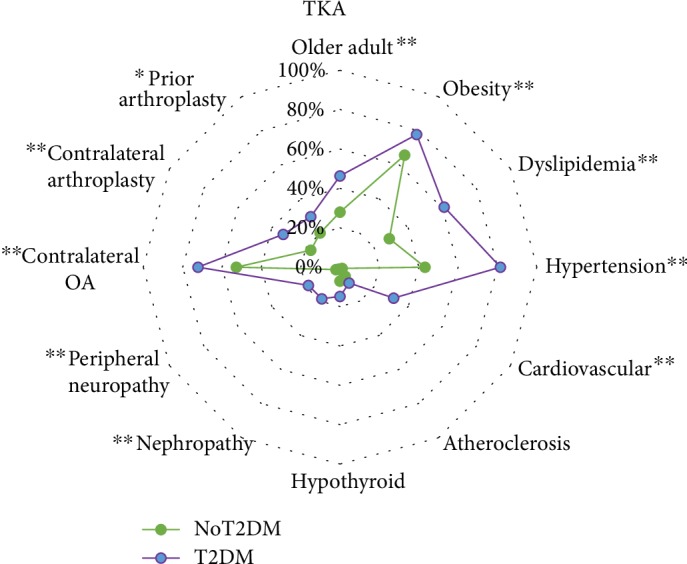
Radar plot showing proportions of individual characteristics, comorbidities, and arthritis per type 2 diabetes mellitus (T2DM) status among those receiving a primary total knee arthroplasty (TKA). Vertices of radar plot represent group proportion of variables for patient characteristics (upper right quadrant of radar plot), comorbidities (bottom half of quadrant), and OA and arthroplasty characteristics of joint involvement (upper left quadrant of radar plot); ^∗^*P* ≤ 0.05; ^∗∗^*P* ≤ 0.01.

Within the TKA cohort, patients with T2DM were older (*P* = 0.01) and had larger BMI (*P* = 0.003) than without T2DM. However, gender (*P* = 0.70), ethnicity (*P* = 0.17), and smoking status (*P* = 0.16) were not significantly different within the TKA cohort between those with and without T2DM (Table 1). Older age accounted for 46.3% of patients with T2DM and 28.0% of patients without T2DM receiving a TKA (Figure 2). Obesity (i.e., BMI ≥ 30 kg/m^2^) was present in 77.8% of the T2DM and 65.7% of the non-T2DM individuals receiving a TKA (Figure 2).

**Figure 3:**
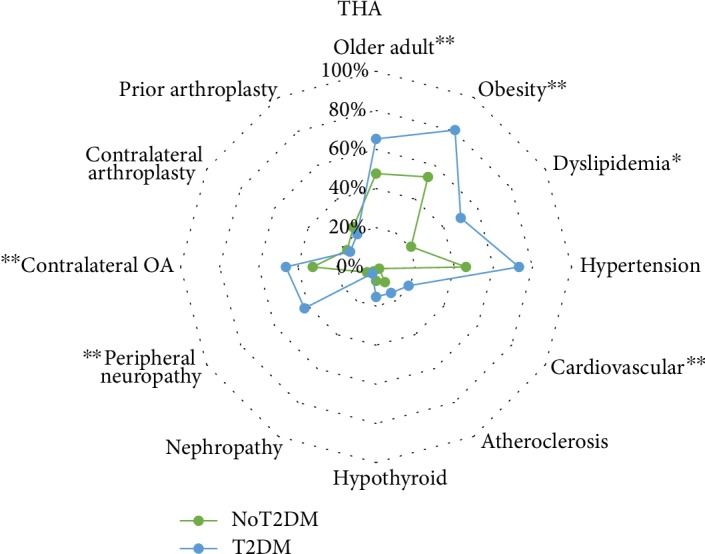
Radar plot showing the proportion of individual characteristics, comorbidities, and arthritis per type 2 diabetes mellitus (T2DM) status among those receiving a primary total hip arthroplasty (THA). Vertices of radar plot represent proportion per group of each variable for patient characteristics (upper right quadrant of radar plot), comorbidities (bottom half of quadrant), and OA and arthroplasty characteristics of joint involvement (upper left quadrant of radar plot); ^∗^*P* ≤ 0.05; ^∗∗^*P* ≤ 0.01.

Within the THA cohort, BMI (*P* = 0.01) was significantly larger for those with T2DM, while age (*P* = 0.14), gender (*P* = 0.25), ethnicity (*P* = 0.67), and smoking status (*P* = 0.11) were not significantly different between those with and without T2DM (Table 1). Older age, or age ≥ 64 years, accounted for 65% of patients with T2DM and 47.7% of patients without T2DM receiving a THA (Figure 3). Obesity (i.e., BMI ≥ 30 kg/m^2^) was present in 81.0% of the T2DM and 53.1% of the non-T2DM individuals receiving a THA (Figure 3).

**Figure 4:**
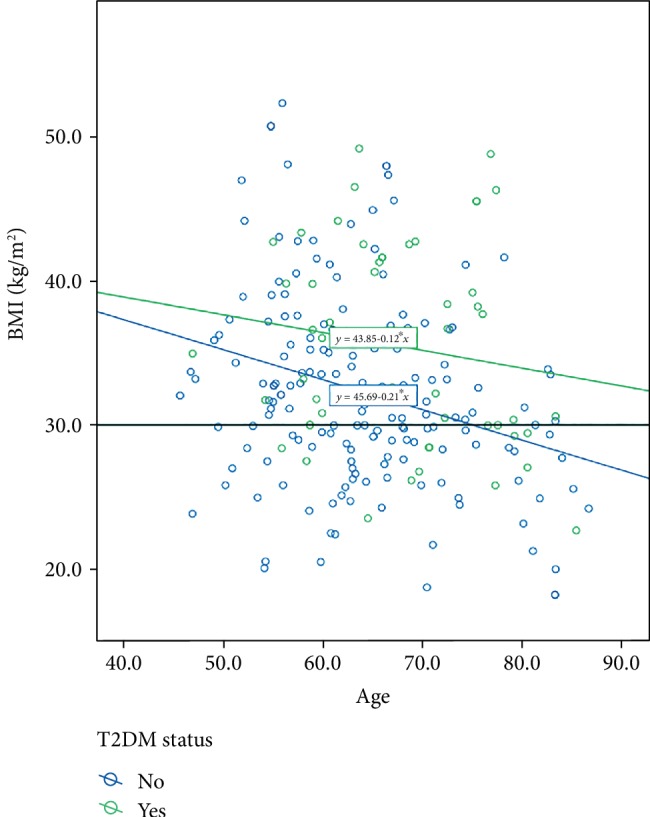
Relationship between body mass index (BMI) and age in years at the time of arthroplasty surgery among patients with and without type 2 diabetes mellitus (T2DM). – indicates cut-off for obesity defined as BMI≥30 kg/m^2^.

The linear relationship between BMI and age at the time of arthroplasty was defined as y = 43.85 − 0.12x for those with T2DM and y = 45.69 − 0.21x for those without T2DM, with *y* = BMI and *x* = age. Therefore, when BMI is relatively similar, patients with T2DM of the same BMI are older (Figure 4).

**Figure 5:**
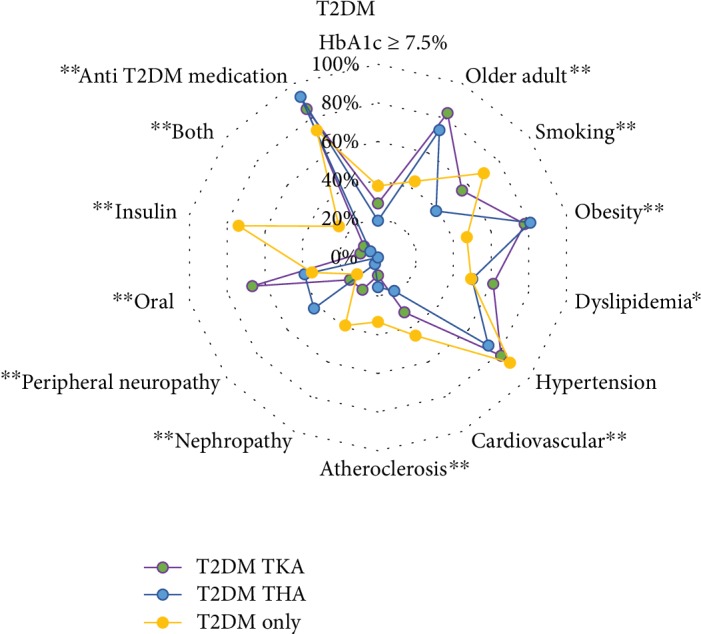
Radar plot showing proportion of individual characteristics, comorbidities, and type 2 diabetes mellitus (T2DM) differences with and without arthroplasty. Vertices of radar plot represent proportion per group of each variable for patient characteristics (upper right quadrant of radar plot), comorbidities (bottom half of quadrant), and OA and arthroplasty characteristics of joint involvement (upper left quadrant of radar plot). TKA = total knee arthroplasty; THA = total hip arthroplasty; ^∗^*P* ≤ 0.05; ^∗∗^*P* ≤ 0.01.

Among the T2DM cohorts, patients with T2DM receiving an arthroplasty, when compared to the T2DM with no-OA group, were older (TKA, mean difference = 8.0, 95% CI [12.7, 3.3], *P* < 0.01; THA, mean difference = 8.4, 95% CI [14.5, 2.3], *P* < 0.01) and had larger BMI (TKA, mean difference = 5.6, 8.5, 2.6, *P* < 0.01; THA: mean difference = 5.1 years, 95% CI [8.8, 1.3], *P* < 0.01) (Table 1). For THA with T2DM, only smoking (TKA, *P* = 0.06; THA: *P* < 0.01) was more common than the T2DM and no OA group. Obesity was present in 47.1% of the T2DM with no-OA group (Figure 5). Meanwhile, older age (i.e., ages ≥ 60 years) was present in 83% of patients with TKA, 73% of those with THA, and 44% of the T2DM with no-OA cohort (Figure 5).

### 3.2. Comorbidities

Of the comorbidities assessed, the TKA patients with T2DM had a greater total number of comorbidities than those without T2DM (*P* < 0.01). With statistical control for age, sex, and BMI, the effect for the group (i.e., T2DM status) yielded statistical significance for urinary (partial *r* = 0.17, *P* < 0.01), cardiovascular (partial *r* = 0.38, *P* < 0.01), nephropathy (partial *r* = 0.31, *P* < 0.01), hypertension (partial *r* = 0.33, *P* < 0.01), dyslipidaemia (partial *r* = 0.25, *P* < 0.01), and peripheral neuropathy (partial *r* = 0.17, *P* = 0.01), but not for atherosclerosis (partial *r* = 0.02, *P* = 0.80) and hypothyroid (partial *r* = 0.10, *P* = 0.09) (Table 2).

The total number of comorbidities reported by the THA group was greater for those with T2DM than without T2DM (*P* < 0.01). After statistical control for age, sex and BMI, the effects of the group were significant for urinary (partial *r* = 0.28, *P* < 0.01), cardiovascular (partial *r* = 0.29, *P* < 0.01), dyslipidaemia (partial *r* = 0.20, *P* = 0.018), and peripheral neuropathy (partial *r* = 0.40, *P* < 0.01), but not for hypertension (partial *r* = 0.158, *P* = 0.067) or nephropathy (partial *r* = 0.02, *P* = 0.779) (Table 2).

Between the T2DM with no OA and the TKA with T2DM patients, the effect of groups with age, sex, and BMI controlled, was significant for cardiovascular (partial *r* = 0.26, *P* < 0.01), nephropathy (partial *r* = 0.27, *P* < 0.01), and atherosclerosis (partial *r* = 0.35, *P* < 0.01), but not for hypertension (partial *r* = 0.11, *P* = 0.18), dyslipidaemia (partial *r* = 0.04, *P* = 0.68), or peripheral neuropathy (partial *r* = 0.28, *P* = 0.03) (Table 2).

Comparisons between the T2DM with no OA and the THA with T2DM groups were significant for the effects of group while controlling for age, sex, and BMI for cardiovascular (partial *r* = 0.30, *P* < 0.01), nephropathy (partial *r* = 0.31, *P* < 0.01), hypertension (partial *r* = 0.22, *P* = 0.02), atherosclerosis (partial *r* = 0.28, *P* < 0.01), and peripheral neuropathy (partial *r* = 0.28, *P* < 0.01), but not for dyslipidaemia (partial *r* = 0.13, *P* < 0.17) (Table 2).

### 3.3. OA and Arthroplasty Characteristics

Group differences between those with and without T2DM receiving an arthroplasty were significant for bilateral OA (partial *r* = 0.17, *P* = 0.01), prior arthroplasty (partial *r* = 0.17, *P* = 0.01), and contralateral arthroplasty (partial *r* = 0.17, *P* = 0.01). However, after controlling for age, sex, and BMI, the group effects on bilateral OA (partial *r* = 0.09, *P* = 0.19), prior arthroplasty (partial *r* = 0.13, *P* = 0.06), and contralateral arthroplasty (partial *r* = 0.12, *P* = 0.07) were no longer significant (Table 3).

### 3.4. T2DM Characteristics

Average HgbA1c levels were not significantly different among the no OA with T2DM and arthroplasty with T2DM groups (Table 4). Arthroplasty and T2DM groups significantly explained antidiabetic medication use and its associated severity (partial *r* = 0.20, *P* = 0.01). Group differences for T2DM duration (*P* = 0.029) were significant; however, post hoc adjustment for multiple comparisons were not significant for comparisons with TKA (mean difference = 3.96 years, 95% CI [-8.32, 0.50], *P* = 0.10) or THA groups (mean difference = 4.67 years, 95% CI [-10.6, 1.3], *P* = 0.180) (Table 4).

### 3.5. Patient Characteristic, DM, and OA/Arthroplasty Relationships

**Figure 6:**
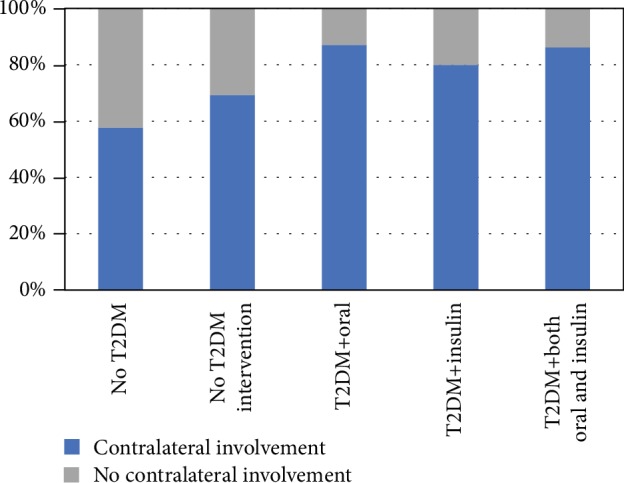
Group breakdown of contralateral joint involvement per type 2 diabetes mellitus (T2DM) and antidiabetic intervention status.

Older adults, or those ≥60 years, with T2DM and TKA or THA requiring antidiabetic intervention, had an OR of 4.5 (95% CI [0.57, 35.83]) for having contralateral joint involvement (i.e., OA or arthroplasty in the contralateral joint) than those with T2DM not requiring antidiabetic interventions (Figure 6).

## 4. Discussion

Approximately 22.8% of adults in the United States have arthritis; however, the prevalence of arthritis for those with T2DM is 54% [15]. Thus, the likelihood of patients with T2DM receiving a TKA or THA due to terminal OA is higher than individuals without T2DM. The current study found that the prevalence of T2DM among patients receiving a primary TKA is 18%, and THA is 24.2%, which is consistent with previous studies. Further, at the time of arthroplasty, patients with T2DM are more likely to be older and obese and characterized by cardiovascular, urinary, dyslipidemia, peripheral neuropathy, and OA involvement in other joints than their arthroplasty non-T2DM counterparts. Of patients with T2DM, individuals pursuing an arthroplasty are older, have larger BMI, report peripheral neuropathy, and use less severe antidiabetic medication, while those with T2DM but and no history of OA are more likely to smoke, have nephropathy, atherosclerosis, and use insulin to manage T2DM. Hence, it appears that patients with T2DM pursuing arthroplasty are the most advanced age and have the largest BMI, which is consistent with previous studies suggesting the metabolic OA phenotype. The result of the current study adds to the growing literature that suggests these individuals are at the highest risk for poor arthroplasty outcomes that require improved care management strategies dictated for this vulnerable population.

A key finding of this study was that patients among the T2DM and arthroplasty cohort were the oldest of all groups, which is consistent with previous studies in those patients with T2DM, who are classified into the metabolic OA phenotype, are older than all other OA phenotypes [16]. Advanced ages are associated with larger risks for pre- and postoperative TKA/THA events, including infection, revision, 30-day readmission, and mortality [17]. Ages 75 years or older at the time of surgery increase the odds of 90-day post-TKA/THA mortality by 2.6-folds compared to those between 65 and 75 years [18]. After 68 years for TKA patients and 63 years for THA patients, postoperative functional gains become worse than those younger [10, 11]. In the current study, approximately half of the T2DM arthroplasty groups exceed this age threshold; therefore, suggesting that age may contribute to the known poor outcomes associated with arthroplasty surgery among patients with T2DM. Perhaps, our current OA care strategy imposes a delay in indicating when primary arthroplasty surgeries need to occur. Among a veteran population, King et al. [19] found individuals with T2DM to be younger than the non-T2DM at the time of their lower extremity arthroplasty. The study differences may be attributed to the health care system and delivery differences. Therefore, careful considerations are needed to improve OA care management that potentially can limit arthroplasty delays for patients with T2DM and OA.

Comorbidities that were specific to the T2DM arthroplasty group included obesity and peripheral neuropathy, which accounted for a more substantial proportion than those receiving an arthroplasty with no T2DM and/or those with T2DM without OA. The role of obesity and its pathophysiological contribution to T2DM and mechanical contribution to OA have been well-established. However, peripheral neuropathy has been considered to a lesser extent and had a significant influence on our study results despite controlling for age and BMI. Peripheral neuropathy was reported among patients with T2DM with almost half of those receiving a THA and 18% of receiving a TKA. Peripheral neuropathy is characterized by sensory degradation and motor degeneration that can alter gait mechanics that either increase loads at the involved joint or redistributes load to adjacent or contralateral joints. T2DM can lead to the denervation of motor neurons [20, 21], infiltration of fat in muscle tissues [22], and reduction in tendon extensibility [23]. Such muscle physiological changes, when combined with neurological changes impacted by diabetic neuropathy, can create altered movement strategies that re-direct weight-bearing forces to joint surfaces that are less capable of withstanding repetitive loading and alter the magnitude of load via impulsive loading [24]. Also, prolonged glucose elevation can cause advanced glycation of the end-product to accumulate at joints causing and progressing joint deterioration.

The relationship between T2DM and OA progression appears to be complex, and its actual mechanism remains unclear and unknown. The current study found that within the T2DM, arthroplasty groups, the older T2DM patients requiring antidiabetic medication were 4.5 times more likely to have OA or arthroplasty in the contralateral joint. Contralateral joint involvement, whether defined as OA or arthroplasty, would suggest more severe OA progression. However, when age, sex, and BMI were statistically controlled contralateral OA, previous arthroplasty or contralateral arthroplasty differences between those with and without T2DM were no longer significant. Therefore, it appears that age and T2DM-specific characteristics may be a specific confounding factor to OA progression that requires further understanding. Specifically, it is unclear if OA progressions were related to worse T2DM severity requiring antidiabetic medication, the biochemistry of medication, and/or the combining factors of T2DM severity and aging and, therefore, requiring additional studies.

This study has several limitations. This study only examined patients whose terminal OA was addressed under the current standards of care. Therefore, patients with uncontrolled T2DM and clinically significant elevated HgbA1c levels who were not cleared for arthroplasty were not included in the study and limit interpretation. The T2DM group with no OA was drawn from a hospital cohort admitted for other medical conditions without a documented history of OA or arthroplasty, which can be at risk for selection bias given the severity of medical attention that was needed for hospitalization. Also, these retrospective database analyses were completed from charts of a single medical center and, in turn, may be less representative of the general population. As with any retrospective analyses, the accuracy of the data relies on the accuracy of medical records, which could not be regulated or evaluated. However, since group comparisons were completed at a single health care center, the same cohort of clinicians performed these routine exams—thereby minimizing variability. It is also recognized that this study only includes patients with terminal OA who met the criteria necessary as deemed by the orthopaedic surgeon for receiving associated TKA/THA, and hospital outcome indicators were not measured. Conversely, the scope of this project was to examine differences between those with and without T2DM undergoing the current standard of practice for terminal OA and associated arthroplasty, which were achieved in this study. It would be beneficial for future studies to examine hospital outcome indicators and management of terminal OA programs for patients with HgbA1c levels greater than 7.5. Hence, this study provides insight and the groundwork required to examine and develop standards of care for terminal OA management among those with T2DM, with the ultimate goal to optimize outcomes for patients with T2DM undergoing TKA or THA.

A better understanding of T2DM among patients undergoing TKA or THA can aid the development of strategies to enhance terminal OA management for musculoskeletal clinicians. We identified a large prevalence of T2DM and associated comorbidities among patients pursuing THA/TKA for terminal OA. Current practice strategies are to wait for Hb1Ac levels to decline before proceeding with arthroplasty; however, HbA1c reduction can take up to 10 years for some and be unattainable for others. Therefore, such practices can be disadvantageous for patients with T2DM who suffer from severe OA symptoms and, in turn, causing, functional disability and physical inactivity when waiting for a TKA/THA. The physical function limitations can have detrimental metabolic and health consequences. Further, the effects of T2DM related comorbidities, specifically, large BMI, dyslipidemia, and peripheral neuropathy can be addressed or attenuated with physical activity and exercise, which are difficult when also combatting terminal OA symptoms. The results of our study can provide the preliminary insight to suggest that current OA and arthroplasty care management are inappropriate for patients with T2DM and/or that T2DM is predisposing arthroplasty-related complications and limited care outcomes.

## 5. Conclusions

The current standard of care for primary TKA or THA management in patients with T2DM appears to be inadequate. Patients with T2DM receiving a primary TKA or THA are more likely to be older, have a higher number of comorbidities, and OA in contralateral OA. Clinicians must consider the risk and benefits of the interaction between the patient's characteristics, risk and presence of comorbidities, OA status of involved and noninvolved joints, and T2DM status when considering appropriate timing of using a TKA or THA to treat OA.

## Figures and Tables

**Table 1:** Group characteristics. Group summary of patients who received a total knee arthroplasty (TKA) and a total hip arthroplasty (THA) grouped by the status of type 2 diabetes mellitus (T2DM). ∗ marks significant differences between T2DM and non-T2DM groups.

	TKA		THA		No OA with T2DM
	T2DM	Non-T2DM	T2DM	Non-T2DM	
Patients, % (*n*)	24.2 (54)	75.8 (169)	19.0 (26)	81.0 (111)	87
Age, mean ± SD (years)	68.7 ± 8.7^a,c^	63.9 ± 9.2^a^	66.8 ± 9.8^c^	63.1 ± 11.6	60.7 ± 12.8^c^
Females, % (*n*)	57.4 (31)	60.4 (102)	57.7 (15)	45.0 (50)	49 (43)
Ethnicity, % (*n*)					
Caucasian	61.1 (33)	69.8 (118)	65.4 (17)	62.2 (69)	48.3 (42)
Black	24.1 (13)	21.9 (37)	19.2 (5)	31.5 (35)	27.6 (24)
Hispanic	14.8 (8)	7.7 (13)	15.4 (4)	6.3 (7)	23.0 (20)
Asian	0 (0)	0.5 (1)	0 (0)	0 (0)	1.14 (1)
BMI, mean ± SD (kg/m^2^)	35.5 ± 6.9^a,c^	32.3 ± 6.8^a^	35.0 ± 6.6^b,c^	31.1 ± 6.8^b^	29.9 ± 7.1^c^
Smoker, % (*n*)	55.6 (30)	44.4 (75)	38.5 (10)^c^	55.9 (62)	70 (62)^c^

*n* = number of patients; SD = standard deviation; BMI = body mass index; ^a^Significant between T2DM versus non-T2DM for TKA cohort; ^b^Significant between T2DM versus non-T2DM for THA group; ^c^Significant between T2DM groups or arthroplasty versus No OA.

**Table 2:** Common comorbidities related to type 2 diabetes mellitus (T2DM).

	TKA		THA		T2DM, no OA
	T2DM	Non-T2DM	T2DM	Non-T2DM	
Urinary, % (*n*)	3.70 (2)^a^	0 (0)^a^	11.5 (3)^a^	0 (0)^b^	—
Cardiovascular, % (*n*)	31.5 (17)^a,c^	1.2 (2)^a^	19.2 (5)^b,c^	1.8 (2)^b^	44.8 (39)^c^
Nephropathy, % (*n*)	18.5 (10)^a,c^	1.2 (2)^a^	3.80 (1)^c^	2.70 (3)	39.1 (34)^c^
Hypertension, % (*n*)	81.5 (44)^a^	43.2 (73)^a^	73.1 (19) ^c^	46 (51)	87.4 (76)^c^
Dyslipidemia, % (*n*)	61.1 (33)^a^	29 (49)^a^	50.0 (13)^b^	20.7 (23)^b^	49.4 (43)
Atherosclerosis, % (*n*)	9.3 (5)^c^	5.3 (9)	15.4 (4)^c^	9 (10)	33.3 (29)^c^
Hypothyroid, % (*n*)	14.8 (8)	7.1 (12)	15.4 (4)	7.2 (8)	—
Peripheral neuropathy, % (*n*)	18.5 (10)^a^	2.4 (4)^a^	42.3 (11)^b,c^	5.4 (6)^b^	13.8 (12)^c^

— indicates that data is unavailable for the T2DM with no OA group. *n* = number of patients; THA = total hip arthroplasty; TKA = total knee arthroplasty; ^a^Significant between T2DM versus non-T2DM for TKA cohort; ^b^Significant between T2DM versus non-T2DM for THA group; ^c^Significant between T2DM groups or arthroplasty versus no OA.

**Table 3:** Osteoarthritis (OA) and arthroplasty characteristics.

	TKA		THA	
	T2DM	Non-T2DM	T2DM	Non-T2DM
Bilateral OA, % (*n*)	72.2 (39)	52.7 (89)	46.2 (12)	32.4 (36)
Prior arthroplasty, % (*n*)	19.6 (16)	20.1 (34)	19.2 (5)	23.4 (26)
Contralateral arthroplasty, % (*n*)	33.3 (18)	17.2 (29)	15.4 (4)	17.1 (19)

*n* = number of patients; T2DM = type 2 diabetes mellitus; THA = total hip arthroplasty; TKA = total knee arthroplasty.

**Table 4:** Type 2 diabetes mellitus (T2DM) characteristics of T2DM patients with the total knee (TKA), hip (THA) arthroplasty, or no osteoarthritis or arthroplasty (No OA) groups.

	TKA	THA	No OA
Hemoglobin A1c, mean (SD)	7.09 (1.1)	6.74 (1.0)	7.6 (2.0)
Anti-diabetic intervention, % (*n*)			
Oral only	66.7 (36)^a^	80.8 (21)^b^	21.8 (19)^a,b^
Insulin only	9.3 (5)^a^	0 (0)^b^	46.0 (41)^a,b^
Both insulin and oral	9.3 (5)^a^	11.5 (3)	16.1 (14)
Unavailable or not on medication for T2DM	14.8 (8)	7.7 (2)^b^	14.9 (13)^b^
T2DM duration, % (*n*), (years)^	11.2 (8.6)^a^	11.9 (8.0)^b^	7.3 (6.4)^a,b^
Duration from T2DM to surgery, % (*n*), (years)^^	10.0 (8.6)	9.2 (7.1)	N/A
Duration of peripheral neuropathy to surgery, % (*n*) (years)	2.6 (1.5)	6.1 (7.3)	N/A

N/A = not applicable; *n* = number of patients; SD = standard deviation; ^a^Significant between TKA and no OA groups; ^b^Significant between THA and no OA groups; ^Duration from T2DM to surgery was available in 18 out of 54 patients with TKA and 9 out of 26 patients with THA; ^^Duration of peripheral neuropathy to surgery was available for all patients with TKA but only in 6 out of the 11 patients with THA reporting peripheral neuropathy.

## Data Availability

The data used to support the findings of this study are available from the corresponding author upon request.
